# Development and Evaluation of a Flexible PVDF-Based Balloon Sensor for Detecting Mechanical Forces at Key Esophageal Nodes in Esophageal Motility Disorders

**DOI:** 10.3390/bios13080791

**Published:** 2023-08-04

**Authors:** Peng Ran, Minchuan Li, Kunlin Zhang, Daming Sun, Yingbing Lai, Wei Liu, Ying Zhong, Zhangyong Li

**Affiliations:** 1 School of Bioinformatics, Chongqing University of Posts and Telecommunications, Chongqing 400065, China; ranpeng@cqupt.edu.cn (P.R.); s210302014@stu.cqupt.edu.cn (K.Z.); sundm@cqupt.edu.cn (D.S.); s220531007@stu.cqupt.edu.cn (W.L.); zhongyingcqupt2023@outlook.com (Y.Z.); 2 Chongqing Engineering Research Center of Medical Electronics and Information Technology, Chongqing 400065, China; lizy@cqupt.edu.cn; 3 School of Optoelectronic Engineering, Chongqing University of Posts and Telecommunications, Chongqing 400065, China

**Keywords:** advanced infusion-compatible balloon catheter, polyvinylidene fluoride (PVDF) piezoelectric sensor matrix, esophageal stress analysis, esophageal biomechanical dynamics

## Abstract

Prevailing methods for esophageal motility assessments, such as perfusion manometry and probe-based function imaging, frequently overlook the intricate stress fields acting on the liquid-filled balloons at the forefront of the probing device within the esophageal lumen. To bridge this knowledge gap, we innovatively devised an infusible flexible balloon catheter, equipped with a quartet of PVDF piezoelectric sensors. This design, working in concert with a bespoke local key-node analytical algorithm and a sensor array state analysis model, seeks to shed new light on the dynamic mechanical characteristics at pivotal esophageal locales. To further this endeavor, we pioneered a singular closed balloon system and a complementary signal acquisition and processing system that employs a homogeneously distributed PVDF piezoelectric sensor array for the real-time monitoring of dynamic mechanical nuances in the esophageal segment. An advanced analytical model was established to scrutinize the coupled physical fields under varying degrees of balloon inflation, thereby facilitating a thorough dynamic stress examination of local esophageal nodes. Our rigorous execution of static, dynamic, and simulated swallowing experiments robustly substantiated the viability of our design, the logical coherence of our esophageal key-point stress analytical algorithm, and the potential clinical utility of a flexible esophageal key-node stress detection balloon probe outfitted with a PVDF array. This study offers a fresh lens through which esophageal motility testing can be viewed and improved upon.

## 1. Introduction

Esophageal motility disorder assessment is a crucial diagnostic tool aimed at evaluating the functional status and coordination of the esophageal wall muscles, which is of significant value in identifying conditions such as esophageal motility disorders, esophageal strictures, and gastroesophageal reflux disease [1]. By measuring and analyzing esophageal pressure, this method can distinguish between various types of motility disorders, including achalasia, diffuse esophageal spasm, and hypertensive lower esophageal sphincter, providing reliable biomechanical information for diagnostic classification and disease progression evaluation. Consequently, the development of novel and efficient esophageal motility disorder detection techniques is of great importance for the treatment of esophageal diseases and clinical research [1,2].

Over the past decade, research on the assessment of esophageal motility disorders has mainly focused on clinical medical issues related to the use of impedance-pressure measurement devices in the diagnosis and treatment of various conditions. This includes a series of medical studies conducted using high-resolution manometry (HRM) and functional luminal imaging probe (FLIP) techniques [3]. Utilizing HRM in conjunction with impedance-pressure measurement has emerged as a prominent approach for assessing esophageal motility disorders [4]. Numerous investigations have underscored the significance of esophageal impedance measurements [5,6], the potential of integrating impedance and manometry techniques [7], as well as the identification of valuable diagnostic parameters [8]. Concurrently, researchers have taken demographic factors into account and devised robust classification criteria [9,10], such as the “Chicago Classification v4.0” (CCv4.0), in order to enhance our comprehension and management of esophageal motility disorders in a more sophisticated manner [2]. The FLIP testing approach employs an infusion–perfusion flexible balloon catheter, deviating from HRM’s infusion–perfusion impedance-pressure detection mode [11,12]. By measuring alterations in diameter, volume, and pressure, it utilizes high-resolution impedance planimetry to generate a three-dimensional esophageal lumen image, primarily focusing on the assessment of esophageal distensibility [13,14]. As impedance planimetry technology advances swiftly, the application of FLIP has become increasingly prominent in the clinical investigation of a diverse array of esophageal disorders, encompassing achalasia [15,16,17], dysphagia [18], EGJ outflow obstruction (esophagogastric junction outflow obstruction) [19,20,21], eosinophilic esophagitis (EoE) [20,21,22], and gastroesophageal reflux disease (GERD) [23,24,25]. These studies underscore the paramount significance of FLIP as a vital tool in the evaluation and understanding of esophageal motility dysfunction within the clinical research landscape [11,14].

However, both HRM and FLIP have limitations, particularly in capturing the strain characteristics of multiple muscle groups in the esophageal region and the stress and strain transmission of various components of the balloon under dynamic micro-deformations. Therefore, to overcome these limitations and improve diagnostic accuracy and patient outcomes, there is a critical need to optimize the front-end device design and data interpretation theories and to develop an intra-balloon flexible pressure sensor that can detect the dynamic mechanical information of different regions of the esophagus. Indeed, the limitations of current HRM and FLIP methodologies, particularly in capturing the strain characteristics of various esophageal muscle groups and stress–strain dynamics of balloon components under micro-deformations, underscore the urgent need for optimization of device design and theory refinement.

To this end, the development of an intra-balloon flexible pressure sensor, capable of discerning dynamic mechanical information from different esophageal regions, emerges as a promising avenue. Emerging technologies, such as gas-sensing capsules utilized in recent studies, have demonstrated potential in accurately identifying critical anatomical landmarks. Although primarily used to measure regional transit times, their working principles offer valuable insights that can inform the design and functionality of our proposed esophageal sensor. The robust inter-observer agreement observed with these gas-sensing capsules and their adeptness at landmark identification indicate potential pathways to enhance the diagnostic accuracy of our envisioned intra-balloon sensor [26,27]. Moreover, the ingestible electronic capsule, capable of sensing gases such as oxygen, hydrogen, and carbon dioxide, presents an innovative approach that could be adapted for esophageal health monitoring. Its ability to modulate and analyze gut microbial fermentative activities via dietary fiber intake manipulation offers a noteworthy perspective for our proposed sensor [27]. This could potentially enhance esophageal region delineation and dynamic mechanical information gathering, providing valuable clinical insights.

Among the available options, piezoelectric PVDF film is a reliable choice for an intra-balloon pressure sensor due to its high sensitivity, resolution, flexibility, and stability [28,29,30]. In biomedical applications such as human signal detection [31,32], strain detection [33], sound sensing [34], and vibration detection [35], PVDF piezoelectric sensors have shown great promise, indicating their extensive potential in flexible pressure detection research. However, challenges in developing the proposed PVDF-piezoelectric-film-based sensor may include technical limitations, manufacturing constraints, and issues related to biocompatibility. To address these challenges, this study aims to design a flexible liquid-filled balloon sensor based on PVDF piezoelectric film for the detection of the mechanical forces at key nodes of the esophageal wall. The study will be conducted from three aspects, including static performance research of the sensor, direct problem research of a stress analysis algorithm based on the data collected by the sensor, and reconstruction of stress at key nodes of the esophageal wall. Both experiments and simulations will be conducted to validate the feasibility and effectiveness of the proposed design, ultimately contributing to a more comprehensive understanding and improved management of esophageal motility disorders.

## 2. Materials and Methods

### 2.1. Stress Detection System Design and Fabrication

**Figure 1:**
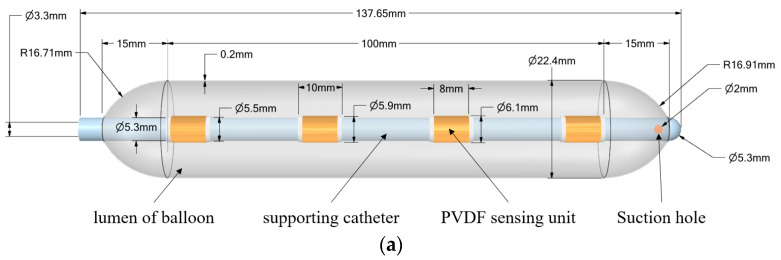

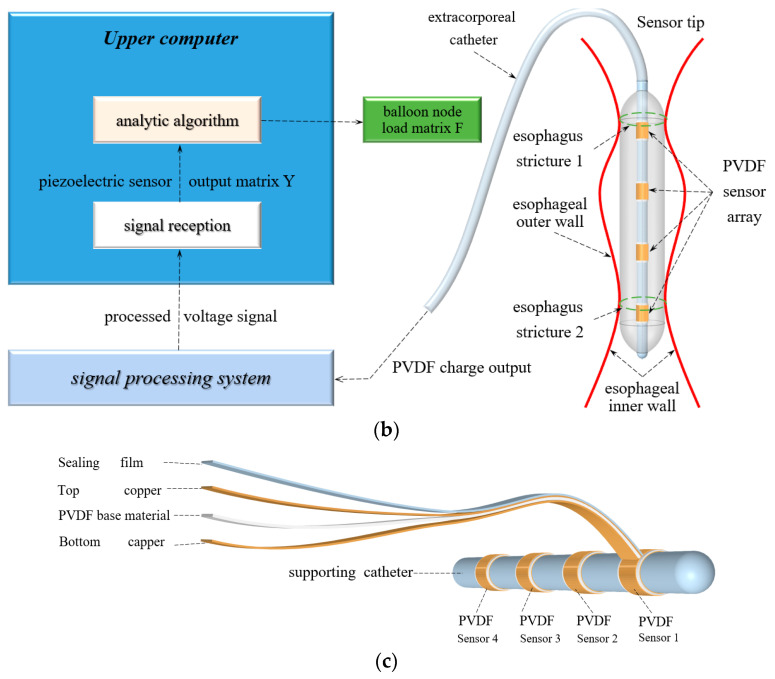
Flexible balloon structure at key points of esophagus based on PVDF: (**a**) schematic diagram of the balloon structure; (**b**) working schematic of the sensor system; and (**c**) PVDF piezoelectric film sensor on the catheter.

In the course of our investigation, we meticulously engineered a flexible, infusible balloon probe, equipped with an array of PVDF piezoelectric sensors. This unique construction is aimed at advancing into the esophagus, thereby enabling accurate detection of dynamic stress at crucial esophageal locations. Figure 1a exhibits a 3D model of the balloon at the forefront of our sensor device. Figure 1b shows a schematic diagram of the whole process of evaluating esophageal motility using this device. Initially, the non-inflated balloon catheter is introduced into the esophagus, followed by gradual inflation to establish a flexible adhesion with the esophageal wall musculature. Subsequently, the external catheter is gently pulled to ensure the balloon evenly traverses a narrowed segment of the esophagus. Concurrently, the signals gathered from the PVDF sensor array are transmitted to the signal processing system via the catheter. Following the filtration, the processed signals constitute a piezoelectric output matrix and are forwarded to an upper computer, where a local key-node analytical algorithm further processes the piezoelectric signals. Ultimately, this generates a balloon node loading matrix that can dynamically represent the dynamic load on the surface of the four local nodes of the balloon. To corroborate the universality of the employed algorithm and experiment, the upper computer system deployed in this investigation was a modest setup operating on a Windows 10 platform. The system comprised an Intel i5 10400 central processing unit (CPU), 8 GB of random-access memory (RAM), and did not possess an independent graphics processing unit (GPU).

It is noteworthy that the production cost of this balloon sensor is relatively low, incorporating core components such as a composite PVDF piezoelectric film, a medical liquid conduction tube, and a medical liquid pouch. During the assembly process, medical-grade solid adhesive (Type 4861) and medical-grade UV adhesive (Type 3321) are employed to tightly seal the PVDF piezoelectric sensors, with closure accomplished by the balloon cavity. The balloon is fabricated from a non-elastic, high-toughness transparent PVC film, primarily comprising a pre-bent hemispherical wall, a post-bent hemispherical wall, and a central cylindrical wall. This ingenious design endows the balloon with the capacity to withstand fluid shear stress and elastic stress without significant deformation.

The overall length of the balloon is 137.65 mm, with a maximum and minimum inner diameter of 22.4 mm and 5.3 mm, respectively, demonstrating its flexibility to accommodate both narrow and non-narrow regions of the esophagus. As depicted in Figure 1c, four PVDF piezoelectric film sensors are evenly distributed along the support tube, each consisting of an upper copper layer, polarized PVDF substrate, lower copper layer, and a medical-grade sealing film for sensor encapsulation. Additionally, medical-grade UV glue and medical sealing film (model-parafilm pm966) are used to tightly wrap and adhere the PVDF sensor assembly to the surface of the tube.

**Figure 2:**
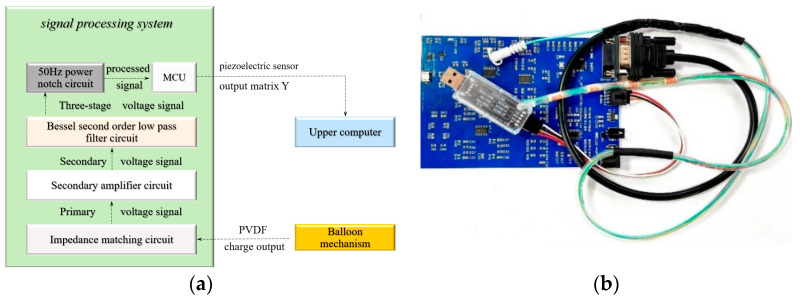
Circuit architecture of esophagus dynamic detection system based on piezoelectric sensor: (**a**) test circuit system architecture and (**b**) sensing device architecture.

The signal processing and control terminal designed as part of this research and adapted to the sensor is mainly composed of a data acquisition and processing module, MCU module, suction and injection liquid control module, and signal transmission module. The schematic diagram of the circuit architecture and the real picture of the complete device are shown in Figure 2a,b, respectively. The core part of the system is a collection and signal processing module composed of a pre-multiplexed charge amplifying circuit, a two-stage multiplex voltage amplifying circuit, a Bezier second-order low-pass filter circuit, and a 50 Hz power frequency notch circuit. 

The external end of the balloon catheter is connected to the pre-amplification circuit through a DB9 needle, and the 4 channels of charge signals collected by the PVDF sensor group are input into the signal detection and processing system at the sampling frequency of 20 Hz. The 4 channels of signal matrix Y [4×20n] obtained at the frequency of *n* Hz within t seconds of the system operation will be sent to the upper position machine through the signal transmission circuit and participate in the stress analytical calculation of balloon joints as an input signal. 

### 2.2. Stress Reconstruction at Key Esophageal Nodes

In this study, we scrutinize the utility and validity of our distinct balloon design using a robust theoretical analysis guided by an innovative esophageal key-node analytical model. This model capitalizes on a dual-process methodology: the forward operation predicting the sensor’s theoretical output from a specific balloon surface load, and the reverse operation inferring the theoretical external load based on a known sensor output. We address algorithmic complexities and practical detection circumstances by confining the model to instances where the load point coincides with the PVDF sensor and focusing on immersed sensors for accurate shear stress computation. The core processes involve dynamic esophageal loading, balloon deformation, and charge generation by the PVDF piezoelectric film in response to elastic strain and fluid shear stress. Adopting quasi-steady-state values for balloon surface load and sensor output, we factor in balloon membrane stress, catheter bending stress, and internal fluid shear stress to derive the sensor’s theoretical output.

To better visualize the intricacies of our methodology, we have encapsulated the entire process in an algorithmic flowchart, ensuring a holistic understanding of the model’s functionalities. This comprehensive model representation is depicted in Figure 1, setting the stage for an in-depth discussion of the full computational algorithm that follows.

#### 2.2.1. Balloon Membrane Surface Stress Calculation

To prevent substantial deformation of the balloon membrane during strain, it was fabricated from an inelastic material devoid of soft hysteresis properties. Throughout the computation and analysis of the mechanical model, the balloon membrane was regarded as a thin shell structure [36,37]. Specifically, the balloon’s membrane structure endures in-plane tensile loads exclusively, and is not subjected to bending loads, thereby exhibiting marginal changes in shape and thickness. Predicated on these assumptions, the surface load ***F_b_*** of the balloon was employed as an input parameter, from which the surface stress of the balloon was derived as follows:

(1) $$\{\begin{array}{c} \sigma_{\theta }=\frac{F_{b}\cdot r}{2h}\\ \sigma_{r}=\frac{F_{b}\cdot r}{h} \end{array}$$

where *σ_r_* is the radial stress along the radius of the balloon, *h* is the thickness of the balloon film, σ_θ_ is the tangential stress along the perimeter of the balloon, and *r* is the radius of the balloon. 

In this study, the stress calculation problem of the balloon film was transformed into the plane stress problem. The surface load of the balloon film when the balloon device was squeezed by the inner wall of the esophagus was solved by the stress equation of classical elastic film material. The equilibrium equation is shown in Equation (2):

(2) $$\frac{\partial \sigma_{r}}{\partial r}+\frac{\left(\sigma_{r}-\sigma_{\theta }\right)}{r}=0$$

The geometric equation is shown in Equation (3):

(3) $$\{\begin{array}{c} \varepsilon_{r}=\partial u/\partial r\\ \varepsilon_{\theta }=\;u/r \end{array}$$

where ***ε_r_*** is the radial strain along the radius of the balloon, ***ε_θ_*** is the tangential strain along the perimeter of the balloon, and *u* is the displacement in the radius of the balloon. Its physical equation is shown in Equation (4):

(4) $$\{\begin{array}{c} \sigma_{r}=\frac{E}{\left(1-v^{2}\right)}\cdot \left(\varepsilon_{r}+v\cdot \varepsilon_{\theta }\right)\\ \sigma_{\theta }=\frac{E}{\left(1-v^{2}\right)}\cdot \left(\varepsilon_{\theta }+v\cdot \varepsilon_{r}\right) \end{array}$$

Here, *E* is Young’s modulus and *v* is Poisson’s ratio. Combined with the above three equations, the relationship between the stress and strain of the balloon film can be derived to further calculate the bending stress of the hose and the shear stress of the fluid in the capsule.

#### 2.2.2. Catheter Bending Stress Calculation

In the esophageal pressure-sensing environment described in this study, the predominant stress component within the balloon cavity structure is radial stress, denoted as *σ_r_*. As the catheter remains unexposed to the esophageal wall during balloon inflation, we simplify the analysis by considering only the bending stress arising from the balloon membrane’s traction on the catheter. Adhering to the *Euler–Bernoulli* beam theory [38,39], the bending stress *σ_bend_* in the catheter can be expressed as Equation 5):

(5) $$\sigma_{bend}=\frac{(\sigma_{r}\cdot A)d\cdot c}{\pi /4(R_{o}^{4}-R_{i}^{4})}$$

Here, *A* represents the cross-sectional area of the catheter, *d* signifies the distance from the cross-sectional center to the balloon axis, *c* corresponds to the maximum distance of the cross-sectional area from the catheter axis, *Ro* denotes the outer radius of the catheter, *R_i_* represents the inner radius of the catheter, and *π* symbolizes the mathematical constant pi.

#### 2.2.3. Intra-Balloon Fluid Shear Stress

The core analysis herein pertains to a balloon device, with its input–output characteristics contingent upon instantaneous static values. This assertion catalyzes the correlation of fluid shear stress inside the balloon to the static *Navier–Stokes* equation [40,41]. Consequently, fluid pressure change, denoted as ∆P, is established via the strain parameters of the balloon film (*ε_r_* and *ε_θ_*) and bulk modulus *K* (where *K* = 2.2 × 10^9^ *Pa* for distilled water), as shown in Equation (6):

(6) $$\begin{array}{l} \Delta P=K\cdot (\varepsilon_{r}+\varepsilon_{\theta }) \end{array}$$

The static *Navier–Stokes* equation, pivotal to this analysis, is formulated with respect to the fluid pressure distribution (*P*), fluid dynamic viscosity (*μ* = 1.002 × 10^−3^ *Pa·s* for distilled water), and the fluid velocity field (*u*), as depicted in Equation (7):

(7) $$\{\begin{array}{c} -\nabla P+\mu \nabla^{2}u=0\\ \nabla \cdot u=0 \end{array}$$

Assuming an axisymmetric flow of the fluid within the balloon aids in simplifying computations, thereby limiting the fluid’s flow velocity components to the radial (*r*) and axial (*z*) directions. The fluid velocity field, represented by *u*, and the radial and axial velocity components are hence expressed in Equation (8):

(8) $$u=\left(\begin{array}{c} u_{r}(r,z),0,u_{z}(r,z) \end{array}\right)$$

Incompressibility of the distilled water stipulates a no-divergence condition for the velocity field, leading to Equation (9):

(9) $$\begin{array}{cc} \nabla \cdot u & =\frac{\partial u_{r}}{\partial r}+\frac{1}{r}\cdot u_{r}+\frac{1}{r}\cdot \frac{\partial (r\cdot u_{\theta })}{\partial \theta }+\frac{\partial u_{z}}{\partial z} \end{array}$$

This analysis reaches a zenith with the computation of fluid shear stress (*τ*) on the hose surface, formulated with the fluid viscosity and the velocity gradient perpendicular to the surface, symbolized as *y*, as explicated in Equation (10):

(10) $$\begin{array}{l} \tau =\mu \cdot \frac{\partial u}{\partial y}|surface \end{array}$$

#### 2.2.4. PVDF Force-Electric Conversion Calculation

Our mechanical model employs four PVDF piezoelectric sensors enveloping the catheter surface, with the surface stress input to a sensor—expressed in Equation (11)—being a synthesis of catheter surface stress (*σ_bend_*), pressure surface stress (*σ_surface_*), and the fluid’s shear stress (*τ*) in the capsule.

(11) $$\sigma_{PVDF}=\sigma_{bend}+\tau +\sigma_{surface}$$

The polarization charge induced on the PVDF surface by *σ_bend_* and *τ* is perpendicular to these stress vectors, with the output charge (*Q_out_*) of PVDF computed as outlined in Equation (12):

(12) $$Q_{out}=d_{31}(\sigma_{PVDF}\cdot s)$$

Here, ‘*s*’ is the sensor’s surface area. Through a bespoke piezoelectric signal detection system, *Q_out_* is converted to output voltage (*V_out_*), as shown in Equation (13):

(13) $$V_{out}=g_{31}(\sigma_{PVDF}\cdot s)$$

Here, *g_31_* is the voltage constant (200 mV/N). This theoretical deduction of the balloon surface load is pivotal in actual esophageal mechanical testing.

#### 2.2.5. Model of Balloon Input–Output Inverse Problem

The 20 Hz piezo output matrix *Y*[4 × 20 n] collected in situ is quasi-statically simplified, neglecting continuous strain and representing the output as a static combination matrix. The balloon node stress static analysis (denoted *P*) reveals the interplay between the sensor output matrix *Y*[4 × 20 n] and the balloon node load matrix *F*[4 × 20 n], as in Equation (14):

(14) $$Y_{\left[4\times 20n\right]}=P\cdot F_{\left[4\times 20n\right]}$$

The inverse of this process (denoted P^−1^) calculates the static stress of key esophageal nodes, represented in Equation (15):

(15) $$F_{\left[4\times 20n\right]}=P^{-1}\cdot Y_{\left[4\times 20n\right]}$$

These calculations generate the theoretical results for local esophageal nodal stress after detection, providing a foundation for further esophageal examinations and evaluations.

### 2.3. Sensor Static Output Characteristic Test

To empirically validate the design of our sensor, comprehensive static and dynamic evaluations were conducted. Due to the absence of a standard reference for pressure detection in dynamic esophageal environments, the sensor’s direct accuracy assessment becomes challenging. Therefore, we resorted to evaluating static performance using sensor sensitivity, linearity, and zero-input response characteristics under various conditions.

Accounting for esophageal strictures in humans, the contact areas between the upper and lower esophageal sphincters, the aortic arch, and our balloon device approximate to 1.77 cm^2^, 4.91 cm^2^, and 4.91 cm^2^, respectively. As direct detection of the load from the esophageal wall onto the balloon film surface is not viable in simulations, we devised an esophageal pressure detection test. This test requires the balloon to be suspended and fixed vertically to minimize gravity-induced errors, while a 2 cm diameter circle serves as the load input. By electronically adjusting the manometer’s load input position and value, we evaluate the sensor’s static characteristics at different fill degrees, providing a basis for assessing the feasibility of our balloon design.

Finally, considering individual variability, the amplitude of the simulated esophageal peristalsis wave for resting conditions is set at 10–30 mmHg, with 20 mmHg as the initial pressure value. The sensor’s pressure detection range is 20 to 150 mmHg, aligning with the esophageal muscle group’s actual load during peristalsis. A 2 cm diameter dome creates the contact interface between the manometer and the balloon, applying a pressure range of 0–4.5 N. For clarification, it is important to note that the ‘N’ referenced in this study merely stands for the standard output values of the pressure application device, employed for ease of experimental operation, and does not depict the actual pressure circumstances. This value can be converted into the standard pressure, denoted in mmHg, by taking into account the pressure contact area on the surface of the sensor balloon. In all subsequent pressure experiments involving the sensor, any pressure represented by ‘N’ will be supplemented with the corresponding converted pressure values in standard units.

**Figure 3:**
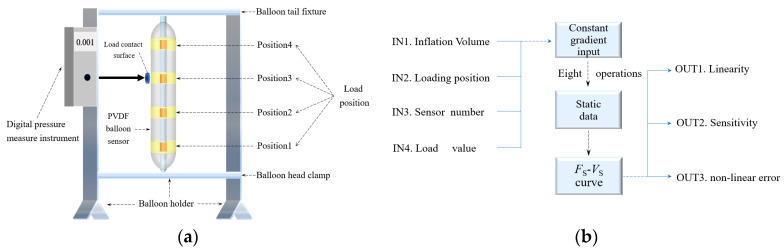
Sensor performance test platform: (**a**) overall structure of the experimental platform and (**b**) static characteristic test procedure of the sensor balloon.

Figure 3b meticulously delineates the static testing methodology for a balloon sensor, comprising five specified fill levels: 0%, 25%, 50%, 75%, and 100%. The exploration is concentrated on a 60–90% optimal detection scope. The balloon’s peripheral surface is judiciously segmented into quadrants (*P1~P4*), each assigned to an inlaid pressure sensor. The assurance of accuracy is strengthened by undertaking eight repetitions for each fill level and location combination. An *F_S_-V_S_* curve, the static calibration curve, is derived via a predetermined load spectrum of [0, 4.5] N ([20, 150] mmHg) at a gradient of 0.045 N (1.3 mmHg). The computation of crucial parameters such as sensor linearity, sensitivity, and zero-input response in the concluding phase consolidates the understanding of the sensor’s static performance features.

Linear regression, applied to the *F_S_-V_S_* curve, yields the linear parameter calculation formula as illustrated in Equation (16):

(16) $$\begin{array}{cc} PVDF_{out} & =K_{s}F_{b}+PVDF_{0} \end{array}$$

where *PVDF_out_*, *K_s_*, *F_b_*, and *PVDF_0_* embody sensor output, linear fitting sensitivity, balloon load input, and zero input response, respectively. The conjunction of these empirical results with theoretical sensor output unveils the linearity degree, delineated in Equation (17).

(17) $$LD=\frac{Max\{PVDF_{out}-PVDF_{tov}\}}{PVDF_{fso}}\times 100\%$$

Here, *LD* represents linearity, *PVDF_out_* is the actual sensor output, *PVDF_tov_* represents the ideal sensor output, and *PVDF_fso_* indicates the full-scale sensor output. Notably, *PVDF_fso_* is the *PVDF_out_* value when F = 4.5 N (150 mmHg), from Equation (17), while *PVDF_tov_* is the output of the balloon node stress analytical algorithm.

Leveraging the linear regression results, Equation (18) captures sensor sensitivity, with St signifying test sensitivity, ∆*PVDF_out_* indicating the minimum input load variation, and *∆F_b_* representing the minimum input gradient difference of balloon load (0.045 N).

(18) $$\left\{ \begin{array}{l} S_{t}=\frac{\Delta PVDF_{out}}{\Delta F_{b}}\\ S_{C}=\sqrt{\frac{1}{2}\left({\left(\frac{1}{n}\sum_{n}^{1}S_{t\_i}\right)}^{2}+K_{s}^{2}\right)} \end{array}\right.$$

The conclusive sensitivity is deduced by contrasting the algorithm’s square root with the average of all specific local sensitivities. This rigorous analysis delivers a comprehensive portrayal of the sensor’s static attributes, facilitating its application in fluid mechanics.

### 2.4. Experimental Methods for Simulating Esophageal Peristalsis

By meticulously manipulating the location and magnitude of extraluminal pressure over time, we simulated two types of dynamic pressure input scenarios. The input load waveform for the critical point pressure detection in the esophagus was a sinusoid with a fixed amplitude, emulating the esophageal peristaltic wave during the swallowing process as a quasi-sinusoidal wave with an amplitude that increases in direct proportion.

#### 2.4.1. Stress Reconstruction at Key Esophageal Nodes

**Figure 4:**
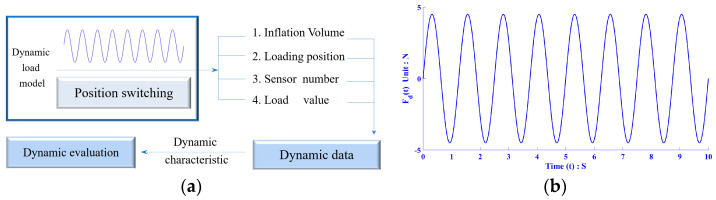
Schematic of dynamic pressure measurement of key nodes: (**a**) key node dynamic pressure measurement process and (**b**) single position sinusoidal load curve.

As depicted in Figure 4a, a consistent sinusoidal load of 4.5 N (150 mmHg) was applied consecutively to four critical nodes of the balloon at five different filling levels. The total testing duration was 40 s, with a sampling frequency of 20 Hz, and a loading time of 10 s for each position.

The sinusoidal load curves for each position are illustrated in Figure 4b. Meanwhile, by incorporating the semi-wave load curves and the temporal variations in the load application positions, the dynamic load application model for critical nodes is expressed in Equation (19).

(19) $$\left\{ \begin{array}{l} F_{d}(t)=4.5\sin(\frac{2\pi }{T_{d}}t)\\ P_{d}(t)=P_{0}+\left\lfloor \frac{t}{T_{d}}\right\rfloor \end{array}\right.$$

Herein, *F_dss_(t)* represents the time-sequenced load value, in units of *N*. *T_d_* denotes the load fluctuation period, and *P_d_(t)* is the load application position. The values 1, 2, 3, and 4 correspond to the critical nodes on the balloon’s outer wall from position1 to position4, respectively. *P_0_* equals 1, indicating the initial position, and $\left\lfloor \frac{t}{T_{d}}\right\rfloor$ is the integer quotient of the time over the load fluctuation period.

#### 2.4.2. Dynamic Output Test under Esophageal Peristalsis during Simulated Swallowing

**Figure 5:**
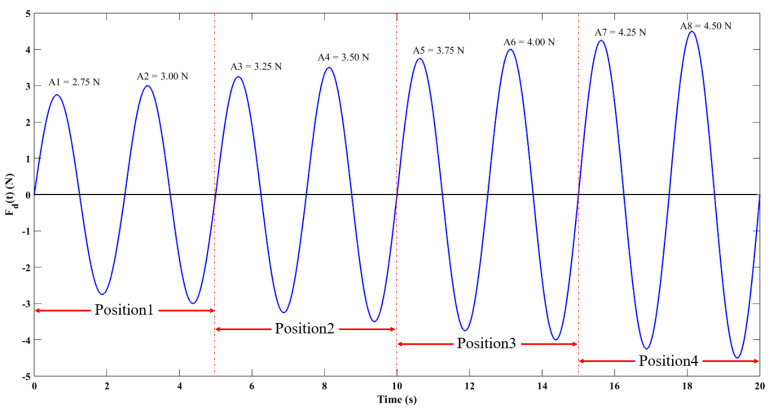
Esophageal peristalsis function curve simulated during swallowing.

The curve representing the esophageal peristaltic wave during the swallowing process is depicted in Figure 5. The detection time was set at 20 s, with a sampling frequency of 20 Hz. To simulate the scenario of a balloon moving at a constant speed, the loading time for each key node was set at 5 s, and the period of variation for the peristaltic wave was fixed at 2.5 s.

The load application function under these conditions is shown in Equation (20).

(20) $$\left\{ \begin{array}{l} F_{C}(t)=F_{0}+A_{n}\sin(\frac{2\pi }{T_{C}}t)\\ P_{C}(t)=P_{0}+\left\lfloor \frac{t}{T_{C}}\right\rfloor \end{array}\right.$$

Herein, *F_c_(t)* is the time-sequenced load value, given in *N*. *T_c_* represents the load fluctuation period, set at 2.5 S, with the total number of fluctuation periods being 8. *P_c_(t)* indicates the load application position, where values 1, 2, 3, and 4 correspond to the critical nodes on the balloon’s outer wall from position1 to position4, respectively. *A_n_* is the load amplitude varying with position. When *P_c_(t)* = [1, 2, 3, 4], the corresponding values are [2.75, 3.00, 3.25, 3.50, 3.75, 4.00, 4.25, 4.50], respectively, after conversion to mmHg, the corresponding values would be [99.44, 106.67, 113.89, 121.11, 128.33, 135.56, 142.78, 150.00]. 

## 3. Results and Discussion

### 3.1. Theoretical Value of Analytical Model of Joint Stress

In the ensuing section, we delve into the results and provide a thorough discussion centered on the theoretical values derived from the analytical model of joint stress. Harmonizing with the International System of Units, we furnish a comprehensive list of the input parameters essential for the algorithmic model, as shown in Table 1. The respective abbreviations and standard units of each parameter follow their names in parentheses, whereas parameters without any units are denoted by “\”.

**Figure 6:**
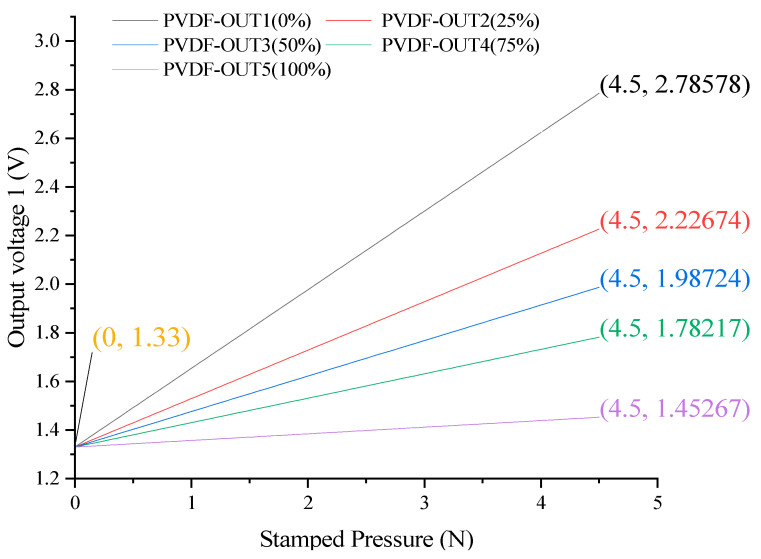
Sensor theoretical output curve.

The instantaneous computation results under a quasi-steady state are characterized as responses when an arbitrary load within the range [0, 4.5]N ([20, 150] mmHg) is applied at a certain position outside the balloon at a particular level of inflation. Using a gradient of 0.045 N (1.3 mmHg), the output values under all loads within the range [0, 4.5]N ([20, 150] mmHg) are plotted to form the theoretical output curves of the sensor, as illustrated in Figure 6. It is important to elucidate that for the purpose of facilitating unit transformation between the sensor material and the circuit system, the Newton (N) unit of the transmuted force will be employed as the representation of pressure in the ensuing outcomes. Redundant restatements of calculation results translated into millimeters of mercury (mmHg) will be circumvented. The correlation between the two aforementioned units has been previously explicated and will exclusively pertain to the scope of the current investigation.

According to the theory of forward process calculation, when the filling degree is 0%, the load will be directly applied to the surface of the PVDF film, and the output voltage will be the largest. At 100% filling, the dynamic flow velocity field cannot be formed, and the surface stress of the PVDF film originates from the catheter bending caused by balloon expansion, and the output voltage is minimal. In other cases, with the increase in filling degree, the fluid shear stress and catheter stress decrease, and the output value and curve slope decrease slightly.

### 3.2. Static Performance Curve and Analysis Results of PVDF Array

To facilitate the discussion, we denote P as a sensor under a specific state feature represented as a vector, as shown in Equation (21):

(21) $$P(n,m,k)\overset{def}{\to }[P_{n},PVDF_{m},k]$$

**Figure 7:**
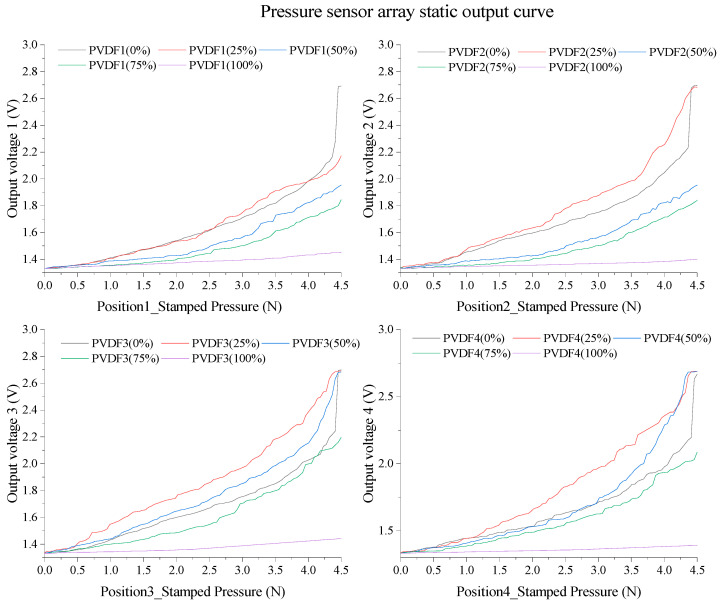
Pressure sensor array static output curve.

Herein, *P_n_*, which encompasses *P_1_*, *P_2_*, *P_3_*, and *P_4_*, represents the pressure loading positions on the outer wall of the balloon, that is, the key nodes Position1 to Position4. The value *m* denotes the sensor number, and both *P_n_* and *m* take the values of 1, 2, 3, and 4. The variable *k* represents the degree of inflation, which can be 0%, 25%, 50%, 75%, or 100%. Figure 7 depicts the static test outputs of the sensor array when loads are applied at different positions under the five types of inflation states of the balloon.

To comprehensively assess the static performance of the sensor across various state characteristics, it is imperative to integrate the static characteristic curve with the previously mentioned static output characteristic table. By linearizing the aforementioned curve, we obtained valuable insights into the linearity, sensitivity, and nonlinear error, as summarized in Table 2. This comprehensive analysis enables us to gain a deeper understanding of the sensor’s static behavior and its associated performance metrics.

**Figure 8:**
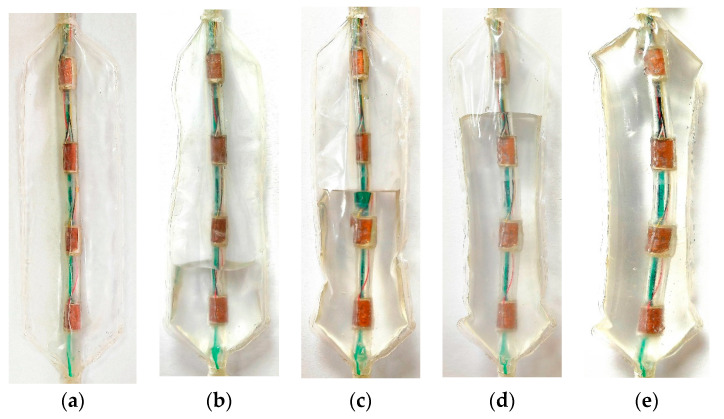
Balloon state under different filling degrees (*k*, %, the amount of fluid filled into the vacuum anhydrous balloon as a percentage of the balloon volume when filled with liquid): (**a**) *k* = 0; (**b**) *k* = 25; (**c**) *k* = 50; (**d**) *k* = 75; and (**e**) *k* = 100.

The balloon states at different filling degrees are shown in Figure 8, and Figure 8a–e represents five cases of 0% to 100% filling, respectively. According to different detection states and experimental results, the state areas of all sensors were divided into vacuum area, fluid attachment area, fast flow field area, slow flow field area, and full filling area, according to the filling degree. As can be seen from the above table, the zero-input response of the sensor in different state regions changes very little, with a mean value of 1.33245 V, while the linearity and sensitivity change obviously. Therefore, the theoretical model, Figure 6, Figure 7 and Figure 8, and Table 2 need to be combined for in-depth analysis to verify the static performance of the sensor.

It is crucial to recognize that due to the impossibility of maintaining an absolute vacuum environment within the balloon throughout the experiment, the vacuum areas discussed herein under various degrees of filling do contain trace amounts of air components. These minimal gaseous constituents pose little interference with the sensor’s pressure testing and are incapable of forming a gaseous domain that could counteract the liquid within the balloon. The thin air present within the balloon is substantially compressed by the dynamic liquid domain. Moreover, the external experimental environment surrounding the balloon is at room temperature and at marked atmospheric pressure. Hence, during the experimental procedure, the fluid domain under consideration pertains solely to the liquid encapsulated within the balloon.

As depicted in Figure 8a, under vacuum conditions, the sensor set encompasses [P(1,1,0), P(2,2,0), P(3,3,0), P(4,4,0)]. In this scenario, the PVDF piezoelectric array exists in a vacuum state with no solution attached to the surface, facilitating the direct transmission of external pressure loads onto the upper copper layer of the PVDF film. This condition results in the maximal sensor output, presenting a sensitivity and linearity of 0.19358 V/N and 28.90955%, respectively, thus providing a baseline for static performance.

Figure 8b–d denotes the fluid adhesion state, where the sensor set comprises [*P(2,2,25), P(3,3,25), P(4,4,25), P(3,3,50), P(4,4,50), P(4,4,75)*]. The adhesive conductive solution in this state collaboratively creates a closed electric field with the sensors, augmenting the conductivity of the sensor surface and consequently elevating the sensitivity to 0.26045 V/N, albeit at a decreased linearity of 16.89788%. Furthermore, in the rapid flow field state (illustrated in Figure 8b–d), the sensor set includes [*P(1,1,25), P(2,2,50), P(3,3,75)*]. Sensors submerged in fluid in this state undergo shear stress, reducing sensitivity to 0.16769 V/N, while linearity is enhanced to 35.96322%.

For the slow flow field state as shown in Figure 8b–d, the sensor set entails [*P(1,1,50), P(1,1,75), P(2,2,75)*]. The buffering effect of the conductive fluid in this state diminishes the shear stress, thereby causing a further decrease in sensitivity to 0.11650 V/N, but a rise in linearity to 44.62696%. Lastly, Figure 8e illustrates the complete filling state, where the sensor set comprises [*P(1,1,100), P(2,2,100), P(3,3,100), P(4,4,100)*]. At this juncture, with an exceptional linearity of sensor static output, sensitivity is reduced to 0.0195325 V/N and linearity drops to 2.99501%, thereby validating the theoretical presupposition that dynamic flow fields are unable to form under complete filling conditions.

### 3.3. Esophageal Creep Simulation Test

The dynamic characteristic experiment uses the same test platform, pressure loading method and analysis model as the static performance experiment. The difference is that the dynamic experiment is to test the overall dynamic output performance of the balloon device and its piezoelectric sensor array in the capsule under the dynamic carrier wave, which is close to the actual esophageal detection environment. It is necessary to consider the case that the load application position does not correspond to the number of the piezoelectric sensor. For example, *P(2,3,75)* is the piezoelectric sensor PVDF3 when the pressure is loaded in Position2 under the filling degree of 75%. Based on the characteristics of dynamic acquisition, the dynamic performance experiments in this study were all performed at the adoption frequency of 20 Hz.

#### 3.3.1. Dynamic Performance Curve and Analysis

**Figure 9:**
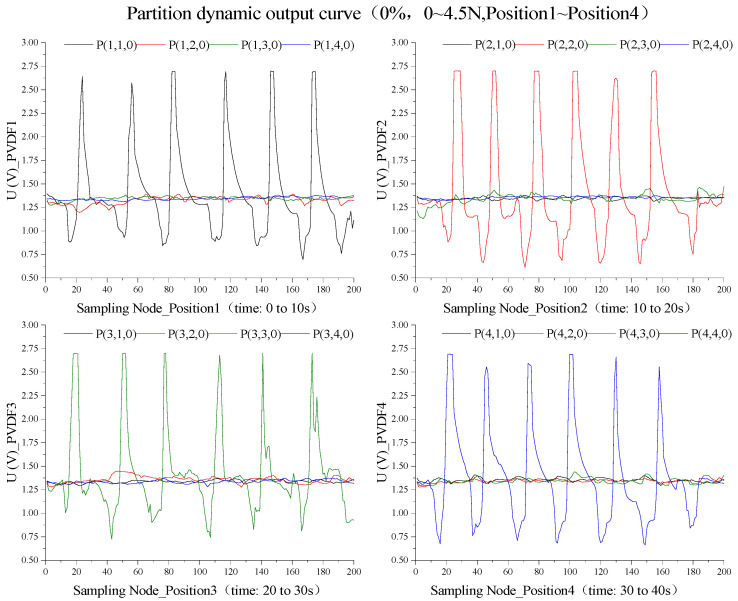
Dynamic output characteristic curve of partition with 0% filling degree.

At *k = 0*, the sensor array inside the capsule occupies a vacuum state, illustrated in Figure 9. The PVDF piezoelectric sensors, under vacuum, exhibit closely matched dynamic full-scale output waveforms with a peak mean of 2.66811 V and a trough mean of 0.77465 V. These sensor dynamics, primarily influenced by manufacturing processes, serve as an important reference for subsequent dynamic tests and esophageal peristaltic wave simulations. By amalgamating static performance results, sensor state definitions, and sensor output model calculations, the dynamic output extremes for various regions are calibrated as [2.78578, 2.22674, 1.98724, 1.45267]V.

**Figure 10:**
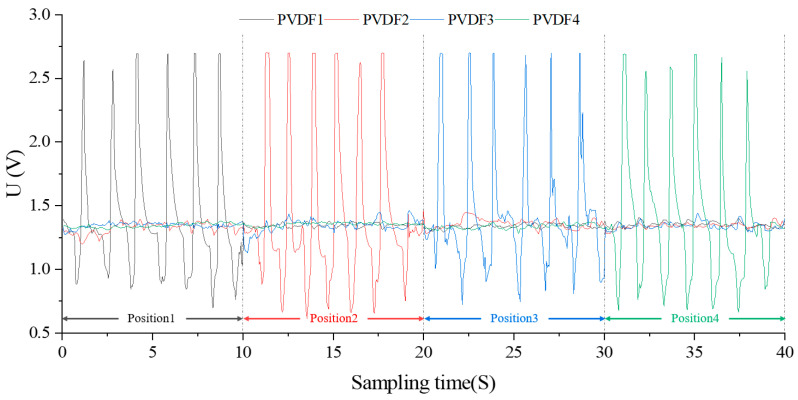
Time series dynamic output curve(*k* = 0).

The hysteresis of piezoelectric materials necessitates load wave cycle adjustments at *k = 0* to attain consistent sensor dynamic output waveforms, which will guide future dynamic experiments. Initial trials confirmed complete output waveforms when six sine load waves of 4.5 N were applied at Positions 1 to 4 over 10 s intervals, with a 20 Hz frequency and 200 sample points per subplot. As shown in Figure 10, this integration resulted in piezoelectric sensor array dynamic output curves in harmony with the input sine load wave features, with a calibration error of −4.4095% for 24 peak mean values.

Considering the hysteresis characteristics of the subsequent reverse charge of the piezoelectric material, it is necessary to adjust the charge-carrier period of *k = 0* to obtain the complete dynamic output waveform of the sensor with good repeatability, which can be used as a reference for the subsequent dynamic experiment. It is preliminarily confirmed by experiments that the output waveform obtained by applying six sinusoidal loaded carrier waves with amplitude of 4.5 N in Position1~Position4 within 10 S is relatively complete. The total time of this experiment is 40 S, the frequency is 20 Hz, and the number of sampling points in each subgraph is 200. After integrating the data in the above image, the obtained dynamic timing output curve of the piezoelectric sensor array is shown in Figure 10. The output waveform of each sensor unit is consistent with the variation characteristics of the input sinusoidal carrier wave, with a total of 24 similar wave peaks, and the calibration error between the average peak and the calibration value is −4.4095%. Among them, the *P(2,2,0)*, *P(3,3,0)*, *P(3,3,0)*, and *P(4,4,0)* test periods are, respectively, [0,10], [10,20], [20,30], and [30,40], and the corresponding pressure load carrier application positions of the four periods are, respectively, Position1, Position2, Position3, and Position4.

**Figure 11:**
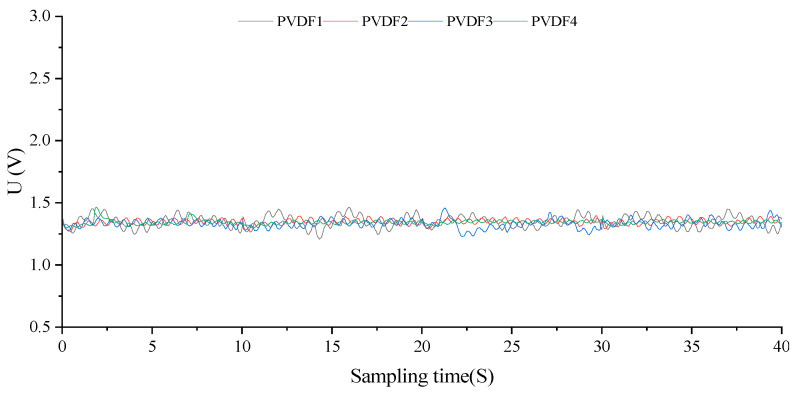
Time series dynamic output curve (*k* = 100).

Upon the attainment of *k = 100*, all sensor arrays transition into a state of full saturation, rendering esophageal mechanics testing unfeasible. As depicted in Figure 11, given the confluence of the capsule’s static hydraulic pressure and the catheter’s mechanical flexure, the emanated waveforms from each sensor closely mirror one another. This mirrored response may serve as a reference for erroneous output under conditions of excessive fluid saturation in esophageal manometry experiments.

**Figure 12:**
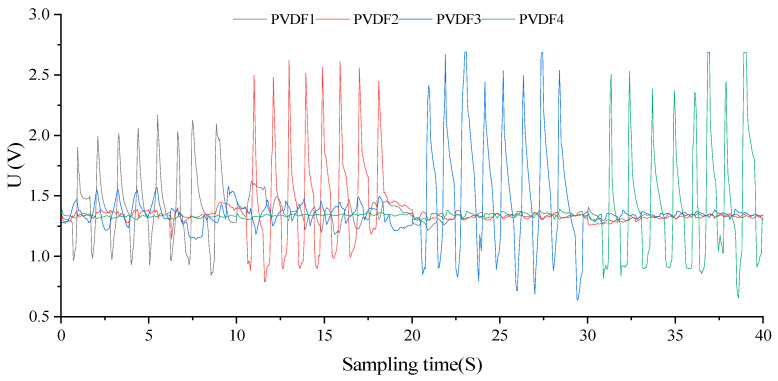
Time series dynamic output curve (*k* = 25).

In an alternative scenario where *k = 25*, the test input sinusoidal charged carrier is different from the standard material performance standard test wave at *k = 0*, but 8 sinusoidal charged carrier waves with amplitude of 4.5 N are applied within 10 S from Position1 to Position4, and the same is true for the input sine wave in the subsequent dynamic test under different filling degrees. At this time, the dynamic time sequence output curve is shown in Figure 12, and its change period is close to the input waveform, which is in the fast flow field area, with an average peak value of 2.04899 V and a calibration error of −8.67500%. *P(2,2,25)*, *P(3,3,25)*, and *P(3,3,25)* are in the fluid attachment area, with an average peak value of 2.66966 V. The calibration error was −4.3496%. Among them, because the dynamic load at Position1 and Position2 will squeeze the solution at the bottom to the position of PVDF3 to form an uneven flow velocity field, the output waveform of *P(1,3,25)* and *P(2,3,25)* is more obvious, and its change period is the same as that of the load carrier, and the average peak is 1.54298 V and 1.46228 V, respectively.

**Figure 13:**
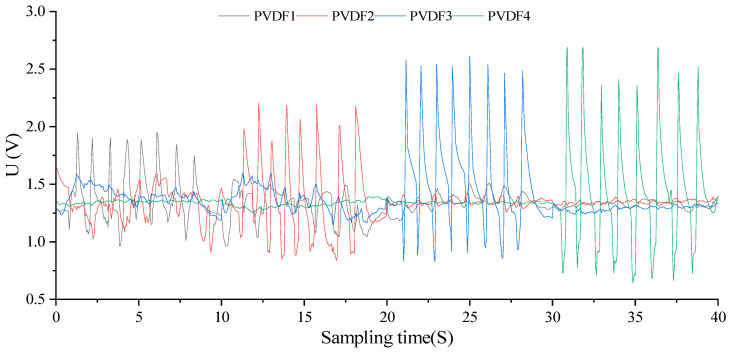
Time series dynamic output curve (*k* = 50).

Upon reaching *k = 50*, as illustrated in Figure 13, the dynamic timing output curve mimics the period of the input waveform. *P(1,1,50)* resides in the slow flow field area, while *P(2,2,50)* is in the fast flow field area, both yielding identical average peaks and calibration errors. Conversely, *P(3,3,50)* and *P(4,4,50)* are situated in the fluid attachment zone, leading to distinct average peaks and calibration errors. Similarly, the dynamic load at Position1 and Position2 induces a displacement of the solution at the base to the position of PVDF3, thereby forming an uneven flow velocity field. Consequently, the output waveforms of *P(1,3,25)* and *P(2,3,25)* are enhanced, with their periodic changes paralleling the load carrier.

It can be seen from the above experimental results that with the continuous increase in filling degree, the dynamic characteristics of the piezoelectric sensor array in the balloon change non-linearly, and the correspondence between filling degree and sensor performance is affected by the complex coupling physical field, so the correlation analysis curve cannot be simply given. However, based on the experience of FLIP experiments and clinical studies, not all balloon filling degrees need to be discussed in detail [12,13,14]. Considering the mechanical properties of the balloon and the fit of the balloon to the esophagus, the filling degree of 65–85% is the ideal filling condition for esophageal function examination [16,17]. Considering the physiological response, the degree of contact between the balloon and the inner wall of the esophagus, and the flow field inside the balloon, the 75% filling level is suitable for the routine mechanical detection of esophageal lymph nodes. In addition, due to the space and experimental limitations of this paper, this study only takes the dynamic experiment under the 75% filling degree as a typical result, but whether 75% is the standard filling degree for the dynamic examination of key nodes of the esophagus needs to be further discussed by subsequent experiments. At the same time, the dynamic experimental results at *k* = 0 in this study only reflect the pressure characteristics of the piezoelectric film in the case of no liquid, which can only be used as the dynamic characteristics of the material but cannot be used as the experimental data support to verify the sensor under the simulated esophageal peristalsis wave.

As such, examining the dynamic characteristics of the sensor array at *k* = 75 is essential for validating the design’s efficacy. At this stage, the sensor array, PVDF1 to PVDF3, is immersed in the solution, resulting in a maximum voltage output of 2.23 V. Given the peristaltic waves during resting and swallowing phases in the esophagus, we defined 1.33 V and 2.23 V as the calibrated output voltages under 0 N and 4.5 N loads, respectively, corresponding to actual esophageal detections at 20 mmHg and 120 mmHg.

**Figure 14:**
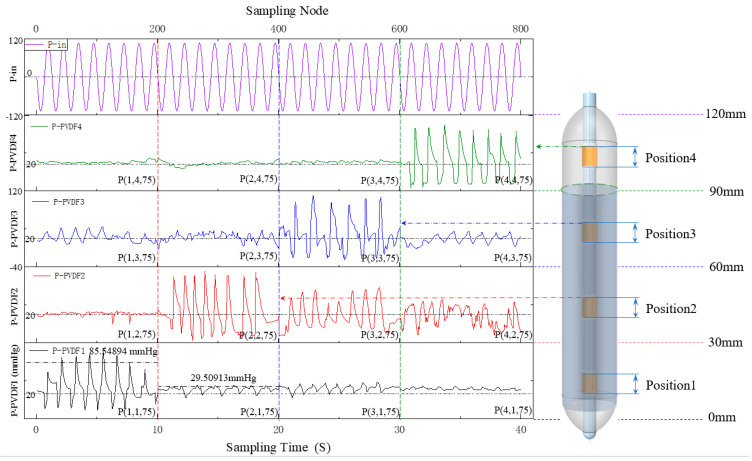
Time–position–pressure curve of the sensor array (*k* = 75).

These detection results were integrated into the analytical model of balloon node stress, culminating in a time-series curve of pressure versus detection time, as illustrated in Figure 14. The left axis of this graph represents pressure values, with a range from −40 mmHg to 120 mmHg for the four sensors, and the zero-input response corresponds to 20 mmHg. To emulate the uniform motion of the balloon in the esophagus, the graph’s right axis denotes load movement distance, with each pressure-loading position set to a range of 30 mm. The output curves of *P(n,n,75)* were subsequently collated based on the detection time.

The average period of the primary output wave of esophageal key point detection (*P_PVDF*) was found to be 1.22574 s, with a standard period error of 1.97921% between P_PVDF and the sinusoidal carrier *P_in_*. The average peak was 94.97334 mmHg, and a standard amplitude error of 20.85556% was noted between *P_in_* and *P_PVDF*. This thorough investigation substantiates the sensor’s proficiency in accurately tracking physiological esophageal behavior.

Apart from the principal output wave, the auxiliary wave provides valuable insight into the dynamic alterations of the balloon surface load. Specifically, the average peak values for *P(2,1,75)* and *P(2,3,75)* are recorded at 30.25399 mmHg and 35.50054 mmHg, respectively. These two output waveforms mirror the node load’s evolving trend at Position1 and Position3 when pressure is applied at Position2. *P(2,1,75)* is situated within the slow flow field and exhibits sensor sensitivity inferior to that of *P(2,3,75)* within the rapid flow field.

Meanwhile, *P(3,1,75)*, *P(3,2,75)*, and *P(3,4,75)* reflect the transformations within the different nodes’ coupled stress field throughout the entire balloon structure when pressure is applied at Position3. *P(3,1,75)* is subject to slow surface fluid flow velocity, heavily influenced by gravity, and yields an average output peak of 38.72979 mmHg. *P(3,2,75)* exhibits a medium surface fluid flow rate under the influence of gravity, resulting in a mean output peak of 63.50647 mmHg. Lastly, *P(3,4,75)* experiences rapid surface fluid flow with a minimal gravitational effect, resulting in an average output peak of 25.09954 mmHg.

Additionally, the average output peaks for *P(4,2,75)* and *P(4,3,75)* are 45.32416 mmHg and 34.6221 mmHg, respectively, aligning with experimental and theoretical characteristics. Collectively, these findings corroborate that the sensor’s output features, as designed in this study, align with the esophagus’ mechanical characteristics and the stress analysis algorithm at key esophageal points under esophageal dynamic manometry experimental conditions. This provides preliminary evidence of the sensor design’s efficacy and applicability in this study and affirms the accuracy of the theoretical algorithm.

#### 3.3.2. Dynamic Analysis under Simulated Esophageal Peristalsis

**Figure 15:**
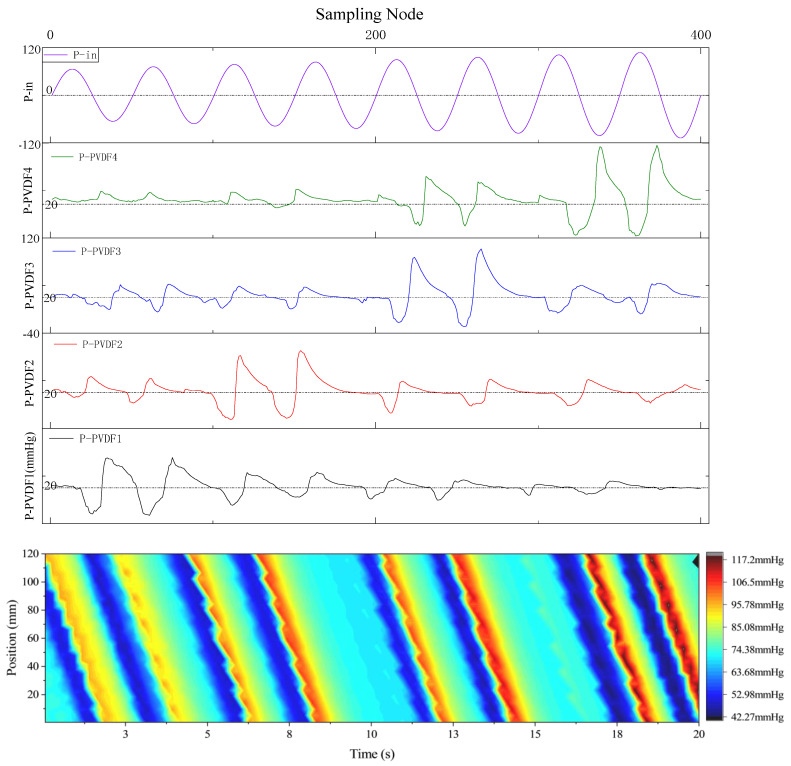
Output curve of sensor array under peristaltic waves simulating the swallowing process (*k* = 75).

The experimental platform and methodology for sensor dynamic output feature detection remain consistent, facilitating the acquisition of the sensor output curve and pressure distribution over time and position under the condition of *k = 75*. This was accomplished during the simulation of the swallowing process of the esophageal peristaltic wave, as illustrated in Figure 15.

With a filling degree of 75%, the simulated esophageal peristalsis wave replicates the extracapsular pressure loading waveform during swallowing, with the amplitude of eight sine waves increasing from 2.75 N to 4.50 N. Utilizing a calibration method akin to the dynamic characteristic experiment, the amplitude of the sensor balloon and pressure loading waveform did not exceed 120 mmHg. Over the four average test periods of [0,20], the dynamic pressure measurement results of the simulated swallowing process were found to align with both the algorithm theory and actual pressure measurements.

The sensor array’s primary waveform output exhibited an average change period of 2.0875 S, with a standard period error of 19.76048%. Compared to the theoretical output of the computational model in this study, the average standard error of the main wave peak in the stress test was measured at 3.33009%. The auxiliary wave’s changing trend concurred with the dynamic detection and analysis findings. From an experimental standpoint, these results verify the balloon sensor and theoretical analysis model’s feasibility designed in this study for practical esophageal motility function detection.

### 3.4. Robustness and Reproducibility

In common esophageal function tests, it is required that external conditions and the physiological state of the human body maintain relative stability. Therefore, the practical application environment for the flexible infusion-style esophageal stress detection balloon based on piezoelectric arrays, which is designed in this study, is predominantly stable. Nevertheless, to counter potential extreme conditions, it is necessary to discuss the robustness and repeatability of the sensor.

Owing to the employment of PVDF piezoelectric films and a medical PVC balloon and catheter, which exhibit remarkably stable mechanical properties, the sensor designed in this study is capable of confronting most extreme strain scenarios. In this study, the sensor underwent rigorous tests, including subjecting it to extreme pressure strains equivalent to two to five times the standard dynamic load, as well as tensile and torsional tests on the sensor’s balloon. As indicated in Table 3, the average error of the standard dynamic test before and after the strain test is presented to denote the mechanical stability of the sensor’s balloon.

Simultaneously, to verify the sensor’s performance stability under extreme external temperature conditions, an interference resistance test based on an external temperature range of 10℃ to 40℃ was designed. The results of this test are illustrated in Table 4. It is worth noting that both sets of tests were based on standard dynamic tests. The displayed results represent the average error between the dynamic test results under specific extreme conditions and the standard dynamic test results under conventional conditions. This error is calculated as the average percentage of error at individual sampling points of the four PVDF piezoelectric sensors’ results, thereby offering a representative insight.

Based on the testing results, it can be inferred that under large strain, the overall performance of the sensor remains temperature-stable, with a minimal dynamic error percentage. With an increase in the force under loading conditions, there is a slight increase in the dynamic error percentage. The smallest and largest error percentages occur post large pressure strain and torsional strain, respectively, with the tensile strain error in the middle range.

Given that the standard dynamic performance test was conducted at 25 °C under standard atmospheric pressure, the error percentage is minimal at 24 °C. Using 24 °C as a critical point, the error percentage slightly increases both with a gradual decrease and increase in temperature. The error percentage under high-temperature conditions is marginally greater than that under low-temperature conditions. The initial validation of the sensor’s robustness, designed in this study, is evidenced by the results from the strain stability test and the temperature stability test.

The static performance and dynamic characteristic tests conducted in this study are based on the average results derived from more than ten repeated experiments. To more rigorously assess the repeatability of the sensor, the ensuing discussion will present the results of 1 to 64 repeated experiments, applying the same error treatment as completed in the robustness test. By adopting the results of 64 repeated experiments as the standard reference, the final results are presented in Table 5.

From the above results, we can observe that as the number of repetitions increases, the percentage of error gradually decreases, highlighting the sensor’s exceptional repeatability.

By associating this insight with the previous robustness test outcomes, it is evident that the percentage of sensor error consistently remains within an acceptable range under varying scenarios. This discovery further validates the sensor’s persistent resilience in confronting extreme strain and temperature perturbations. In essence, even amidst such fluctuating environmental conditions, the sensor persistently maintains the stability of its dynamic output properties, thereby revealing its excellent robustness and repeatability. Conclusively, these comprehensive findings underscore the sensor’s potential for reliable and consistent performance in diverse and challenging conditions, thereby affirming its suitability for complex real-world applications.

## 4. Conclusions

This study presents and validates an innovative, flexible, inhalable liquid closed balloon equipped with a PVDF piezoelectric sensor array. The device was developed to measure the real-time dynamic mechanical characteristics of four critical nodes in the esophageal segment during esophageal function examination. By scrutinizing the coupled physical field of the balloon at various filling levels, a comprehensive analytical model was created, factoring in capsular strain, conduit strain, the fluid flow field, and the piezoelectric film force-electric coupling field.

Three experimental designs were conducted to assess the balloon device’s static and dynamic performance and the dynamic output under simulated esophageal peristaltic wave. The sensor array’s state was differentiated into five regions (vacuum, fluid attachment, rapid and slow flow fields, and full filling) corresponding to the balloon’s filling degree. Experimental outcomes established the sensor’s efficacy in aligning with the theoretical expectations across all filling degrees and validated the stress analysis algorithm for esophageal function testing.

However, it is recognized that further enhancements are feasible. Primarily, using a superior PVDF piezoelectric film material would augment the detection accuracy and range. Secondly, the finite element method could be employed to enable more comprehensive theoretical verification. Lastly, integrating the balloon structure with other detection methods, such as esophageal endoscopy and impedance detection, could facilitate multimodal information detection and fusion analysis, providing richer insights into esophageal motility function.

## Figures and Tables

**Table 1:** Parameter values input into the algorithm model.

Parameter\Filling Degree	0%	25%	50%	75%	100%
1. Elasticity modulus (*E*, MPa)	20	20	20	20	20
2. Calculated diameter of balloon (*R*, mm)	0.5	5.6	11.2	16.8	22.2
3. Balloon thickness (*h*, mm)	0.2	0.2	0.2	0.2	0.2
4. Poisson’s Ratio (*v*, \)	0.35	0.35	0.35	0.35	0.35
5. Diameter of load surface (*d_n_*, mm)	15	15	15	15	15
6. Inner tube radius (*R_i_*, mm)	3.3	3.3	3.3	3.3	3.3
7. Outer tube radius(*R_o_*, mm)	5.0	5.0	5.0	5.0	5.0
8. Distance to the central axis of the balloon membrane in the filled state (*d*, mm)	45	45	45	45	45
9. Tube cross-sectional area (*A*, mm^2^)	11.07	11.07	11.07	11.07	11.07
10. Volume modulus (*K*, MPa)	2200	2200	2200	2200	2200
11. Fluid dynamic viscosity (*μ*, cP)	1.5	1.5	1.5	1.5	1.5
12. Piezoelectric coefficient (*g_31_*, mV/N)	0.2	0.2	0.2	0.2	0.2
13. PVDF surface area(*s*, cm^2^)	1	1	1	1	1
14. PVDF Zero Input Response (*PVDF_0_*, V)	1.33	1.33	1.33	1.33	1.33

**Table 2:** Static performance table of sensor array.

Steady-State Performance\Filling Degree	0%	25%	50%	75%	100%
1. Linearity_PVDF1(*LD*_P1, %)	29.59382	36.4666	46.11891	44.44342	1.38517
Sensitivity_PVDF1(*S_C_*_P1, V/N)	0.18967	0.18496	0.13494	0.10848	0.02607
Zero-input_PVDF1(*PVDF_0_*_P1, V)	1.33055	1.33015	1.33096	1.33015	1.33015
2. Linearity_PVDF2(*LD*_P2, %)	27.63658	21.69366	46.32882	43.31854	4.02154
Sensitivity_PVDF2(*S_C_*_P2, V/N))	0.19472	0.27715	0.13105	0.10608	0.01425
Zero-input_PVDF2(*PVDF_0_*_P2, V)	1.33203	1.33337	1.33305	1.34124	1.33015
3. Linearity_PVDF3(*LD*_P3, %)	28.31165	15.49573	20.0427	25.09424	2.12105
Sensitivity_PVDF3(*S_C_*_P3, V/N))	0.19786	0.29016	0.26229	0.18705	0.02494
Zero-input_PVDF3(*PVDF_0_*_P3, V)	1.33337	1.3374	1.33096	1.33096	1.33015
4. Linearity_PVDF4(*LD*_P4, %)	30.09618	16.30085	23.40208	30.11328	4.45227
Sensitivity_PVDF4(*S_C_*_P4, V/N))	0.19206	0.29136	0.28059	0.16042	0.01287
Zero-input_PVDF4(*PVDF_0_*_P4, V)	1.33176	1.3374	1.33176	1.33257	1.33096

**Table 3:** Robustness test (extreme strain).

Loading Condition\Filling Degree	0%	25%	50%	75%	100%
1. Pressure_2 times (%)	0.81545	0.94521	1.10525	1.21594	\
Pressure_3 times (%)	0.99452	1.08452	1.18752	1.28751	\
Pressure_4 times (%)	1.23015	1.43541	1.59627	1.65873	\
Pressure_5 times (%)	1.69854	1.85463	1.93309	2.10548	\
2. Tensile_2 times (%)	1.56248	1.86326	1.96247	2.05478	\
Tensile_3 times (%)	1.76236	1.89631	1.99632	2.18236	\
Tensile_4 times (%)	1.89544	1.93325	2.16548	2.36514	\
Tensile_5 times (%)	1.95421	2.15659	2.35623	2.59874	\
3. Torsion_2 times (%)	1.71219	1.89654	1.96587	2.16594	\
Torsion_3 times (%)	1.90548	1.95631	2.14856	2.23658	\
Torsion_4 times (%)	2.10585	2.15698	2.19658	2.37892	\
Torsion_5 times (%)	2.43289	2.56612	2.69878	2.72364	\

**Table 4:** Robustness test (Extreme external temperature).

Temperature\Filling Degree	0%	25%	50%	75%	100%
1. Temperature _10 °C (%)	2.41202	1.93325	2.16548	2.36514	\
2. Temperature _12 °C (%)	2.25456	1.89631	1.99632	2.18236	\
3. Temperature _14 °C (%)	2.19455	1.86326	1.96247	2.05478	\
4. Temperature _16 °C (%)	2.11483	2.19878	2.30154	2.41254	\
5. Temperature _18 °C (%)	1.98457	2.15664	2.18965	2.32549	\
6. Temperature _20 °C (%)	1.82365	1.92345	1.98544	2.08934	\
7. Temperature _22 °C (%)	1.65452	1.78953	1.85412	1.92302	\
8. Temperature _24 °C (%)	0.659514	0.79862	0.84512	0.98754	\
9. Temperature _26 °C (%)	1.14854	1.23144	1.25478	1.28656	\
10. Temperature _28 °C (%)	1.28563	1.37894	1.48951	1.56334	\
11. Temperature _30 °C (%)	1.36578	1.54872	1.66891	1.89541	\
12. Temperature _32 °C (%)	1.64214	1.89651	1.99842	2.14523	\
13. Temperature _34 °C (%)	1.85415	2.05998	2.25486	2.32786	\
14. Temperature _36 °C (%)	2.08654	2.30154	2.48965	2.55483	\
15. Temperature _38 °C (%)	2.28124	2.51243	2.64877	2.89654	\
16. Temperature _40 °C (%)	2.68872	2.87541	2.98545	3.15568	\

**Table 5:** Repeatability test.

Times of Repetition\Filling Degree	0%	25%	50%	75%	100%
1. 1 time (%)	4.23124	4.92346	5.25442	5.85621	\
2. 2 times (%)	3.86042	4.56211	4.98847	5.13234	\
3. 4 times (%)	3.24427	3.98551	4.26591	4.86214	\
4. 8 times (%)	2.87563	3.15478	3.45337	4.23668	\
5. 16 times (%)	2.12567	2.56448	2.89314	3.54120	\
6. 32 times (%)	1.42588	1.59846	2.04518	2.13354	\
7. 64 times (%)	\	\	\	\	\

## Data Availability

Data are available upon request by contacting the corresponding author.
